# Typing of hemotropic Mycoplasma in Egyptian cats: first detection and phylogenetic analysis of *Candidatus* Mycoplasma turicensis

**DOI:** 10.1007/s11259-025-10693-0

**Published:** 2025-03-11

**Authors:** Mahmoud S. Safwat, Noha M. Bakry, Amany D. Bahr, Ahmed Orabi, Asmaa A. Rayan, Ghada M. Khalil, Salma W. Abdelhaleem, Omnia H. Refaei

**Affiliations:** 1 Department of Medicine and Infectious Diseases (Infectious Diseases), Faculty of Veterinary Medicine, Cairo University, Giza, 12211 Egypt; 2 Department of Internal Medicine and Infectious Diseases (Infectious Diseases), School of Veterinary Medicine, Badr University in Cairo (BUC), Badr City, 11829 Egypt; 3 Department of Microbiology and Immunology, Faculty of Veterinary Medicine, Cairo University, Giza, 12211 Egypt; 4 Department of Microbiology and Immunology, Faculty of Veterinary Medicine, Assuit University, Assuit, 71526 Egypt; 5 Department of Clinical Pathology, Faculty of Veterinary Medicine, Cairo University, Giza, 12211 Egypt

**Keywords:** Egypt, Feline hemotropic mycoplasma, Phylogenetic analysis, Prevalence

## Abstract

*Mycoplasma haemofelis* (Mhf), *Candidatus* Mycoplasma haemominutum (CMhm), and *Candidatus* Mycoplasma turicensis (CMt) are the major feline hemotropic *Mycoplasma* (FHM) species identified in cats worldwide. Data concerning FHM in Egypt is limited; therefore, the current study aimed to gain further insights into disease epidemiology by investigating FHM molecular prevalence, risk factors, and hemogram abnormalities in 246 Egyptian cats (shelter-housed and client-owned) during 2022–2024. Additionally, *16 S rRNA* gene-based maximum-likelihood (ML) phylogenetic analysis was performed for all CMt-positive samples for the first time in Egypt. FHM was detected in 16.3% (*n* = 40) of cats and typed as CMhm, Mhf, and CMt in 15.4% (*n* = 38), 3.6% (*n* = 9), and 2.4% (*n* = 6) of cats, respectively. Single CMhm and Mhf infections were detected in 12.6% (*n* = 31) and 0.8% (*n* = 2*)* of cats, respectively. Dual (Mhf and CMhm) and triple (Mhf, CMhm, and CMt) infections were found in 0.4% (*n* = 1) and 2.4% *(n* = 6*)* of cats, respectively. CMhm was significantly associated with single infections rather than coinfections compared to other FHM species. Male sex and anemia were identified as predictors of FHM PCR positivity. FHM-infected cats had significantly lower hematocrit %, RBC counts, and hemoglobin concentrations than non-infected ones. Egyptian CMt sequences showed 97.6–100% nucleotide identity with each other. Egyptian and reference CMt strains represented nine nucleotide sequence types clustered into three well-supported clades on the ML tree without clear geographic distinction. The data generated in this study, conducted in Egypt, is crucial for enhancing our understanding of disease epidemiology and implementing effective preventive measures.

**Supplementary Information:**

The online version contains supplementary material available at 10.1007/s11259-025-10693-0.

## Introduction

Feline hemotropic Mycoplasma (FHM) refers to small, pleomorphic, cell wall-lacking, epicellular bacteria found on feline erythrocytes (Tasker et al. 2018). Based on *16 S rRNA* gene analysis, three FHM species (*Mycoplasma haemofelis* (Mhf), “*Candidatus* Mycoplasma haemominutum” (CMhm), and “*Candidatus* Mycoplasma turicensis” (CMt) have been identified worldwide (Barker 2019).

FHM is more prevalent in intact male cats, particularly those with outdoor access lifestyles, and in stray and shelter-housed cats than in client-owned ones, supporting the role of aggressive interactions between cats in disease transmission (Attipa et al. 2017; Kamrani et al. 2008; Sarvani et al. 2018; Willi et al. 2006a). Additionally, cats in geographic areas with warm weather may exhibit higher disease prevalence, suggesting vector involvement, particularly fleas (Willi et al. 2006a), though this transmission route remains debated (Moore et al. 2024).

The overall FHM prevalence across various geographic areas is 9–30%; CMhm has the highest prevalence, ranging from 4.4 to 46.7%, followed by Mhf, with a prevalence ranging from 0.4 to 27.0%, while CMt exhibits the lowest prevalence, ranging from 0 to 26.0% (Tasker et al. 2018). Most prevalence studies were conducted in the Americas, Asia, Australasia, and Europe, whereas only a few have been performed in the Middle East and Africa (Ceylan et al. 2024a, b; Moore et al. 2024).

The primary clinical manifestation of FHM-associated disease is regenerative hemolytic anemia; however, icterus and marked reticulocytosis are uncommon (Tasker et al. 2018). Cats infected with Mhf often exhibit the most severe clinical disease regardless of immune status; in contrast, cats exposed to CMhm and CMt rarely show clinical disease unless they are immunosuppressed or coinfected with more than one FHM species (Sarvani et al. 2018).

FHM diagnosis can be attempted by cytological examination of stained blood smears and molecular techniques, including conventional and real-time PCR (Tasker et al. 2018). Cytology is no longer recommended due to its low sensitivity and specificity; moreover, it cannot detect CMt or differentiate between CMhm and Mhf (Cetinkaya et al. 2016). Molecular techniques are considered the gold standard for diagnosing these uncultivable bacteria, offering accurate diagnosis and FHM species differentiation (Barker 2019). Nonetheless, cats exposed to previous infections may remain positive for variable periods, complicating the diagnostic process (Willi et al. 2006a).

The epidemiological investigation of FHM in Egypt, like in other Middle Eastern and African countries, is a critical research area. At the start of this study (Dec 2022), no published data existed regarding the prevalence, risk factors, hematological abnormalities, or molecular characterization of FHM in Egyptian cats. A recent study reported an overall FHM prevalence of 20% in a convenience sample of client-owned cats in Egypt, identifying CMhm and Mhf in 18% and 2% of cases, respectively; neither CMt nor any form of coinfections was detected (Zarea et al. 2023).

The present study aimed to provide comprehensive data on FHM molecular prevalence in cats (client-owned and shelter-housed) randomly sampled across a wide geographic area in Egypt. Furthermore, it aimed to offer the first molecular characterization and phylogenetic analysis of Egyptian CMt strains.

## Materials and methods

### Sample size calculation, study population, and sampling

The sample size statistically required to estimate FHM prevalence in Egyptian cats was calculated using the following equation: $\:n=\frac{\text{Z}^2\text{P}(1\:-\:\text{P})}{\text{d}^2}$, where n = sample size, Z = Z-statistic for a confidence level (CL), P = expected prevalence, and d = margin of error (Naing et al. 2022). Z was set as 1.96 (95% CL), P was determined as 0.2 based on a recent Egyptian study (Zarea et al. 2023), and d was selected as 0.05. Accordingly, the required sample size was 246.

The study population consisted of 246 cats, including 202 shelter-housed and 44 client-owned cats, recruited from different rescue shelters and veterinary clinics, respectively, in three Egyptian governorates (Cairo, Giza, and Al-Qalyubia) from December 2022 to April 2024. Cats were randomly selected using the probability systematic method; in shelters, the first cat’s cage was chosen by the simple random method, and the remaining cats’ cages were selected at certain intervals, while in clinics, cats admitted on a particular day each month were selected (Thrusfield et al. 2017). Data regarding age, sex, neutering status (males), health status, and housing condition of cats were recorded.

Blood (4 mL) was collected from the jugular vein and divided into two EDTA vacutainers. Within 24 h of sampling, blood was transferred on ice to the Faculty of Veterinary Medicine, Cairo University (FVMCU). One EDTA vacutainer was stored at −20 °C, pending molecular investigations, while the other vacutainer underwent immediate hematological examination.

## Molecular detection and typing of feline hemotropic Mycoplasma

Genomic DNA was extracted from 200 µL of EDTA-whole blood samples using the QIAamp^®^ DNA Blood Mini Kit, following the manufacturer’s protocol. At the start of this study, DNA-positive controls for different FHM species were not available to perform molecular investigations. To obtain positive controls, ten cats from the study population, suspected of being infected with FHM based on clinical examinations, hematology, and cytology results, were tested using FHM species-specific PCR techniques and sequencing. Three positive samples were obtained and used as positive controls for Mhf (accession no. PQ328756), CMhm (accession no. PQ328758), and CMt (accession no. PQ328759) throughout the study period.

All blood samples were screened for FHM DNA using a conventional PCR methodology that detects any FHM species (Jensen et al. 2001). Negative samples were tested for GAPDH (internal control) (Furtado et al. 2017), while positive samples were subjected to three FHM species-specific PCR methodologies to determine the implicated species (Santos et al. 2009; Watanabe et al. 2003). All FHM PCR methodologies partially amplify the *16 S rRNA* gene. The specificity, primer sequences, expected amplicon size, and annealing temperature of all PCR methodologies used in this study are provided in Table 1. No template control (nuclease-free PCR water) was used during DNA extraction and PCR to monitor contamination.

**Table 1:** The specificity, primers sequences, expected amplicon size, and annealing temperature of all PCR methodologies used in this study

PCR method (specificity)	Primers’ sequence (5’–3’)	Amplicon size	Ta
FHM Screening^a^ (all FHM spp.)	for-ACGAAAGTCTGATGGAGCAATA	170 bp (Mhf & CMt) 193 bp (CMhm)	60 °C
	rev-ACGCCCAATAAATCCGGATAAT		
FHM species-specific^b^ (Mhf)	for- ATGCCCCTCTGTGGGGGATAGCCG	275 bp	58 °C
	rev-ATGGTATTGCTCCATCAGACTTTCG		
FHM species-specific^b^ (CMhm)	for- CTGGGAAACTAGAGCTTCGCGAGC	202 bp	58 °C
	rev-ATGGTATTGCTCCATCAGACTTTCG		
FHM species-specific^c^ (CMt)	for-GTATCCTCCATCAGACAGAA	488 bp	55 °C
	rev-CGCTCCATATTTAATTCCAA		
Internal control^d^ (GAPDH)	for-GCCGTGGAATTTGCCGT	164 bp	60 °C
	rev -GCCATCAATGACCCCTTCAT		

Abbreviations: *CMhm* *Candidatus* Mycoplasma haemominutum; *CMt* *Candidatus* Mycoplasma turicensis; *FHM *feline hemotropic mycoplasma; *Mhf* *Mycoplasma haemofelis*; *T(a) *annealing temperature

^a^Jensen et al. 2001

^b^Watanabe et al. 2003

^c^Santos et al. 2009

^d^Furtado et al. 2017

## Hematological investigations

The hemogram of all cats was analyzed using the automated cell counter ABC Vet (ABX Diagnostics, Montpellier, France). The hemogram included the following parameters: hematocrit (HCT) as %, red blood cell (RBC) count as ×10^6^/µL, hemoglobin (Hb) concentration as g/dL, mean corpuscular volume (MCV) as femtoliters (fL), mean corpuscular hemoglobin concentration (MCHC) as g/dL, and absolute reticulocyte count as ×10^6^/µL. Cats were considered anemic if their HCT values were < 30% (Aiello and Moses 2016).

### Partial sequencing and phylogenetic analysis of CMt *16 S rRNA* gene

All samples testing positive for CMt-specific PCR were molecularly characterized by partial *16 S rRNA* gene sequencing. The amplicons were purified from the agarose gel using the QIAquick^®^ PCR Purification Kit (Qiagen, Germany) and sequenced directly with the PCR primers and the BigDye Direct Cycle Sequencing Kit (ThermoFisher, USA) according to the manufacturer’s protocols.

The sequences generated in this study (*n* = 6) were aligned with reference sequences (*n* = 32), including sequences deposited in GenBank (accessed in October 2024) that matched the length and genomic position of our sequences and represented different countries, periods, and hosts (Table 2), using the BioEdit software. CMt sequences obtained in this study were deposited in GenBank under the following accession numbers: PQ328757, PQ328759, and PQ328760-PQ328763. Egyptian and reference CMt sequences were grouped into several nucleotide sequence types (ntSTs), each containing identical sequences. The identity matrix table of different ntSTs was generated by BioEdit software. The phylogenetic relationship between the Egyptian and reference strains of CMt was inferred using the Maximum Likelihood (ML) statistical method. The Kimura-2 parameter nucleotide substitution model was selected as the best model fitting our dataset for the ML analysis. The ML phylogenetic tree was constructed, and the reliability of its branches was assessed by bootstrap tests (1000 replicates). Mhf (EU145745) and CMhm (EU285281) partial *16 S rRNA* gene sequences were added as outgroups. All evolutionary analyses were conducted in MEGA software version 11.

**Table 2:** The name, country of origin, host, and accession number of references *Candidatus* Mycoplasma turicensis *16 S rRNA* gene sequences used in this study

Country of origin	Strain name	Host	Accession number
Australia	D1	*Felis catus*	DQ464417
Australia	B3	*Felis catus*	DQ464423
Australia	D9	*Felis catus*	DQ464425
Brazil	22	*Leopardus pardalis*	DQ825448
Brazil	Porto Alegre	*Felis catus*	EU861063
Brazil	Porto Alegre 1	*Felis catus*	EU580598
Brazil	Porto Alegre 2	*Felis catus*	EU580599
Brazil	F3	*Felis catus*	KM275262
Brazil	clone CAT22	*Felis catus*	KC970340
Brazil	B1	*Felis catus*	KM275258
Brazil	B2	*Felis catus*	KM275259
Brazil	G4	*Felis catus*	KM275266
Brazil	G5	*Felis catus*	KM275267
France	4	*Felis silvestris*	DQ825449
France	10	*Felis silvestris*	DQ825450
Italy	IT226	*Felis catus*	EU839977
Latvia	C60	*Felis catus*	MG456680
South Africa	A11	*Felis catus*	DQ464418
South Africa	C12	*Felis catus*	DQ464419
South Africa	G5	*Felis catus*	DQ464422
South Africa	D7	*Felis catus*	DQ464424
Switzerland	The prototype strain	*Felis catus*	AY831867
Switzerland	CMt clone 2.24	*Felis catus*	DQ157150
Switzerland	CMt clone 946.3	*Felis catus*	DQ157151
Switzerland	CMt clone 365102.5	*Felis catus*	DQ157152
Switzerland	CMt clone 76660.3	*Felis catus*	DQ157153
Switzerland	CMt clone 408606.3	*Felis catus*	DQ157154
Taiwan	66	*Felis catus*	JQ689949
Taiwan	93	*Felis catus*	JQ689950
Thailand	BK-366	*Felis catus*	EU789559
UK	57 A	*Felis catus*	DQ464420
UK	108 N	*Felis catus*	DQ464421

### Statistical analysis

All statistical analyses were conducted using IBM SPSS (version 27) and online biostatistics tools (https://www.openepi.com and https://astatsa.com/FisherTest). The overall prevalences of FHM, CMhm, Mhf, and CMt and the prevalence of various infection types (single and combined, i.e., dual or triple infections) were calculated alongside 95% confidence intervals (CIs). Differences between FHM species regarding prevalence rates and infection types were assessed using the Pearson chi-square or Fisher exact test, with statistical significance indicated by a *P*-value < 0.05. The odds ratio (OR) and its 95% CI were calculated, with 0.5 added to all cells when one cell contained zero.

The association between overall FHM or single CMhm infection rates and selected risk factors, including age (≤ 6 years vs. > 6 years), sex, neutering status of males (intact vs. castrated), housing condition (shelter-housed vs. client-owned), and anemic status (anemic vs. non-anemic), was analyzed using univariate logistic regression. Factors with a *P*-value < 0.2 were selected as candidates for the final multivariable logistic regression model, with statistical significance considered at *P* < 0.05. Additionally, OR and 95% CI were calculated.

Continuous hemogram variables were tested for distribution normality using the Shapiro-Wilk test. Normally distributed variables were presented as mean ± standard deviation (SD), while non-normally distributed variables were expressed as medians and interquartile ranges (IQR). Three cat groups (overall FHM-infected, single CMhm-infected, and FHM non-infected) were compared for hemogram variables using ANOVA for normally distributed data and the Kruskal-Wallis test for non-normally distributed data. Statistical significance between group pairs was determined by the post-hoc Dunn’s test, with a *P*-value < 0.05.

## Results

### Prevalence and typing of feline hemotropic mycoplasma in the study population

The overall prevalence of FHM and each species, and the prevalence of various FHM infection types are summarized in Table 3. All FHM PCR-negative samples tested positive for GAPDH, ruling out inadequate DNA extraction. The supplementary Fig. 1 shows ethidium bromide stained-agarose gel pictures of amplicons of the PCR methodologies used in this study.

**Table 3:** The overall prevalence of FHM and each species, and the prevalence of various FHM infection types (single and combined), including 95% CI, in the study population

	No. of positive cats^a^	Prevalence %	95% CI^b^
Overall FHM^c^	40	16.26	11.65, 20.87
Overall CMhm^d^	38	15.4	10.93, 19.96
Overall Mhf ͩ	9	3.6	1.31, 6.00
Overall CMt^d^	6	2.4	0.51, 4.37
Various infection types^e^			
CMhm (single infection)	31	12.6	8.46, 16.75
Mhf (single infection)	2	0.8	0.10, 2.91
CMhm + Mhf (dual infection)	1	0.4	0.01, 2.24
CMhm + Mhf + CMt (triple infection)	6	2.4	0.51, 4.37

Abbreviations: *CI *confidence interval; *CMhm* *Candidatus* Mycoplasma haematominutum; *CMt* *Candidatus* Mycoplasma turicensis; *FHM *feline hemotropic Mycoplasma; *Mhf* *Mycoplasma haemofelis*

^a^The total number of cats examined in this study is 246 cats

^b^The 95% CIs of all prevalences were calculated by the Wald normal approximation method except for Mhf single infection and CMhm + Mhf dual infection which were calculated by the Clopper-Pearson “exact” method since np̂ were < 5

^c^ Overall FHM means infection with any hemotropic Mycoplasma species

^d^Prevalence of each FHM species included single and combined infections

^e^Single infection with CMt and dual infection with either CMhm and CMt or Mhf and CMt were not recognized in the study population

CMhm showed a significantly higher detection rate compared to Mhf (*P* < 0.001; OR = 4.8. 95% CI 2.2–10) and CMt (*P* < 0.001; OR = 7. 95% CI 3–17.6). The prevalence rate of Mhf did not differ significantly from that of CMt (*P* = 0.43). CMhm was significantly associated with single infections rather than coinfections compared to Mhf (*P* = 0.0014; OR = 15.5. 95% CI 2.6–91) and CMt (*P* < 0.001; OR = 54. 95% CI 2.7–1080). No significant difference was detected between Mhf and CMt regarding infection types (single vs. coinfection) (*P* = 0.48).

## Feline hemotropic mycoplasma-associated risk factors

Table 4 summarizes the distributions of overall FHM-infected and single CMhm-infected cats, including statistical significance, based on specific characteristics of the study population. In the final multivariable logistic regression model, overall FHM PCR positivity was statistically higher in males compared to females (*P* < 0.001; OR = 10. 95% CI 3.3–30.6) and in anemic cats compared to non-anemic ones (*P* = 0.002; OR = 3.5. 95% CI 1.5–8). Regarding single CMhm-infected cats, only male sex was identified as a risk factor in the univariate logistic regression (*P* < 0.001; OR = 8.2. 95% CI 2.4–28).

**Table 4:** The distribution of overall FHM-infected and single CMhm-infected cats, including statistical significance, according to different characteristics of the study population

Infection type^a^	Variable	Categories	*n*	Positive			Univariate analysis			Multivariate analysis		
				*n*	%	95% CI	*P* ^b^	COR	95% CI	*P* ^c^	AOR	95% CI
Overall FHM infection^d^ (*n* = 40)	Housing conditions	Shelter-housed	202	35	17.3	12.1–22.5	0.33	1.6	0.6–4.4	-	-	-
		Client-owned	44	5	11.4	3.7–24.5						
	Sex	Male	142	36	25.3	18.2–32.5	**<0.001**	**8.5**	**2.9–24.7**	**<0.001**	**10.16**	**3.3–30.6**
		Female	104	4	3.8	1.05–9.55						
	Neutering status	Intact	57	16	28	16.4–39.7	0.54	1.2	0.6–2.7	-	-	-
		Castrated	85	20	23.5	14.5–32.5						
	Age	≤6 years	111	12	10.8	5.03–16.5	**0.16**	1.6	0.8–3.3	0.41	1.37	0.6–2.9
		>6 years	135	22	16.2	10–22.5						
	Anemic status	Anemic	55	15	27.2	15.5–39	**0.014**	**2.5**	**1.2–5.1**	**0.002**	**3.5**	**1.5–8**
		Non-anemic	191	25	14.8	9.5–20.2						
Single CMhm infection (*n* = 31)	Housing conditions	Shelter-housed	202	28	13.8	9–18.6	0.21	2.2	0.6–7.5	-	-	-
		Client-owned	44	3	6.8	1.4–18.6						
	Sex	Male	142	28	19.7	13.1–26.2	**<0.001**	**8.2**	**2.4–28**	-	-	-
		Female	104	3	2.8	0.6–8.2						
	Neutering status	Intact	57	16	28	16.4–39.7	0.74	1.1	0.5–2.6	-	-	-
		Castrated	85	12	14.1	6.7–21.5						
	Age	≤6 years	111	11	9.9	4.3–15.4	0.25	1.6	0.7–3.4	-	-	-
		>6 years	135	20	14.8	8.8–20.8						
	Anemic status	Anemic	55	9	16.3	6.5–26.1	0.34	1.5	0.6–3.4	-	-	-
		Non-anemic	191	22	11.5	6.9–16.						

Abbreviations: *AOR *adjusted odds ratio; *CMhm* *Candidatus* Mycoplasma haemominutum; *CI *confidence interval; *COR *crude odds ratio; *FHM *feline hemotropic Mycoplasma

^a^Risk factor analyses were not conducted for other types of infection, including single Mhf and CMt infection, and dual or triple infections due to the absence or small number of cats in these groups

^b^Overall FHM infection means infection with any FHM species

ͨOnly factors with a *P* value of < 0.2 in the univariate logistic regression are suitable candidates for multivariable analyses as shown in bold

^d^Factors with a *P* value of < 0.05 in the final multivariable logistic regression model were considered significantly associated with infection as shown in bold

## Feline hemotropic Mycoplasma-associated hemogram abnormalities

The mean ± SD or median and IQR of different hemogram parameters for overall FHM-infected, single CMhm-infected, and FHM non-infected cats are presented in Table 1. Compared to the control group, overall FHM-infected cats had significantly lower HCT (*P* = 0.006) and Hb concentration means (*P* = 0.01), as well as a lower RBC count median (*P* = 0.01); however, all values remained within the reference range (low normal values). Single CMhm-infected cats did not exhibit statistically significant differences in any hemogram parameter compared to either overall FHM-infected or FHM-non-infected cats.

**Table 5:** The hemogram of FHM infected and control cat groups

Hemogram parameters (measurement unit)	Ref. range^a^	Overall FHM group (*n* = 40)	CMhm-single group (*n* = 31)	Control group (*n* = 206)	*P* value
		Mean ± SD^b^ or Median (IQR) ͨ	Mean ± SD^b^ or Median (IQR)^c^	Mean ± SD^b^ or Median (IQR)^c^	
HCT (%)	30–45	31.92 ± 8.86^d^	33.22 ± 8.72	35.98 ± 8.39^d^	**0.006**
Hb conc (g/dL)	9.8–15.4	10.50 ± 3.53^e^	10.9 ± 3.31	12.06 ± 3.46^e^	**0.016**
RBCs (10⁶/µL)	5.0–10.0	7.67 (5.32)^f^	8.3 (4.75)	9.00 (3.90)^f^	**0.016**
MCV (fL)	39–55	41.25 (14.83)	40.2 (13.5)	39.74 (12.64)	0.24
MCHC (g/dL)	30–36	31.59 (7.39)	31.4 (9.39)	32.27 (3.90)	0.6
Retic. (10³/µL)	< 60	45 (0.12)	32 (0.08)	30 (0.055)	0.33

The hemogram of FHM infected and control cat groups

Abbreviations: *CMhm* *Candidatus* Mycoplasma haemominutum; *FHM* feline hemotropic Mycoplasma; *fL* femtoliter; *HCT* hematocrit; *Hb* hemoglobin; *IQR* interquartile range; *MCHC* mean corpuscular hemoglobin concentration; *MCV* mean corpuscular volume; *RBCs* red blood cells; *Retic* Reticulocytes; *SD* standard deviation

^a^Reference (Aiello and Moses 2016)

^b^Means ± SD were calculated for normally distributed hemogram parameters (HCT and Hb) and were tested statistically by the ANOVA test

^c^Medians and IQR were calculated for NOT normally distributed hemogram parameters (RBCs count, MCV, MCHC, reticulocyte count) and were analyzed statistically by the Kruskal-Wallis test

^d e f^The post-hoc Dunn’s test denoted a significant difference between groups with the same superscript letter at a *P* value of < 0.05

### Partial sequencing and phylogenetic analysis of CMt *16 S rRNA* gene

**Fig. 1:**
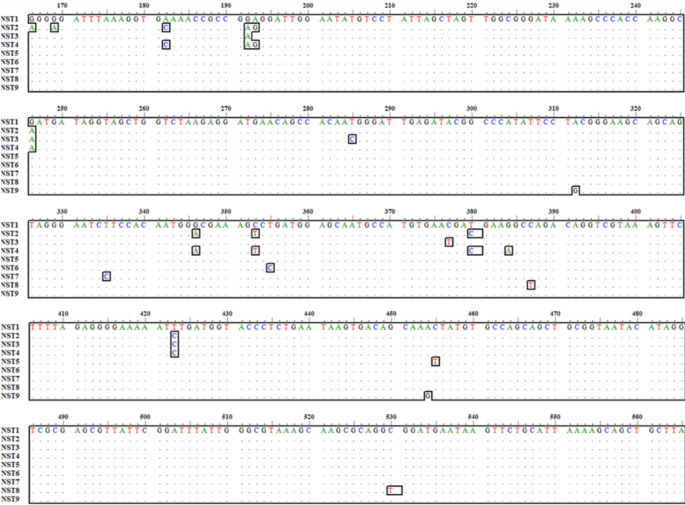
Single nucleotide polymorphisms of nine different *Candidatus* Mycoplasma turicensis (CMt) nucleotide sequence types (ntSTs) of partial *16s rRNA* gene (425 bp) (position numbers are related to the genomic sequence of the prototype strain, accession no. AY831867) ntST 1 (*n* = 18) includes Prototype strain-Switzerland (AY831867), and strains 043/23-Egypt (PQ328759), 118/23-Egypt (PQ328757), 155/23-Egypt (PQ328760), 278/24-Egypt (PQ328761), 226-Italy (EU839977), 93-Taiwan (JQ689950), D1-Australia (DQ464417), 57 A-UK (DQ464420), 108 N-UK (DQ464421), CMt clone 946.3-Switzerland (DQ157151), CMt clone 365102.5-Switzerland (DQ157152), 22-Brazil (DQ825448), 4-France (DQ825449), 10-France (DQ825450), Porto Alegre 1-Brazil (EU580598), and F3-Brazil (KM275262); ntST 2 (*n* = 12) includes 160/23-Egypt (PQ328762), 296/24-Egypt (PQ328763), B3-Australia (DQ464423), D7-South Africa (DQ464424), D9-Australia (DQ464425), Porto Alegre 2-Brazil (EU580599), CMt clone CAT22-Brazil (KC970340), B1-Brazil (KM275258), B2-Brazil (KM275259), G4-Brazil (KM275266), G5-Brazil (KM275267), and C60-Latvia (MG456680); ntST 3 (*n* = 2) includes CMt strains G5-South Africa (DQ464422), and 66-Taiwan (JQ689949); ntST 4 (*n* = 1) includes CMt strain BK-366-Thailand (EU789559); ntST 5 (*n* = 1) includes CMt clone 2.24-Switzerland (DQ157150); ntST 6 (*n* = 1) includes CMt strain C12-South Africa (DQ464419); ntST 7 (*n* = 1) includes CMt clone 76660.3-Switzerland (DQ157153); ntST 8 (*n* = 1) includes CMt strain A11-South Africa (DQ464418); ntST 9 (*n* = 1) includes CMt clone 408606.3-Switzerland (DQ157154). Egyptian sequences generated in this study are indicated by red color

All CMt-specific PCR-positive samples were successfully sequenced, producing six CMt partial *16 S rRNA* gene sequences, each 425 nucleotides in length. The Egyptian CMt strains exhibited 97.6–100% nucleotide identity with each other and 97.1–100% nucleotide identity with the 32 reference CMt strains. The partial Egyptian and reference CMt sequences were grouped into nine ntSTs. The identity matrix table and single nucleotide polymorphisms of different CMt ntSTs are presented in Table 5 and Fig. 1, respectively.

**Table 6:** Nucleotide identity matrix table of nine different *Candidatus* Mycoplasma turicensis (CMt) nucleotide sequence types (ntSTs) of partial *16s rRNA* gene (425 bp) including nucleotide positions 166 to 590 (positions are referred to the genomic sequence of the prototype strain, accession no. AY831867)

	ntST 1	ntST 2	ntST 3	ntST 4	ntST 5	ntST 6	ntST 7	ntST 8	ntST 9
ntST 1	ID	0.976	0.988	0.978	0.997	0.997	0.997	0.995	0.995
ntST 2	0.976	ID	0.978	0.992	0.974	0.974	0.974	0.971	0.971
ntST 3	0.988	0.978	ID	0.981	0.985	0.985	0.985	0.983	0.983
ntST 4	0.978	0.992	0.981	ID	0.976	0.976	0.976	0.974	0.974
ntST 5	0.997	0.974	0.985	0.976	ID	0.995	0.995	0.992	0.992
ntST 6	0.997	0.974	0.985	0.976	0.995	ID	0.995	0.992	0.992
ntST 7	0.997	0.974	0.985	0.976	0.995	0.995	ID	0.992	0.992
ntST 8	0.995	0.971	0.983	0.974	0.992	0.992	0.992	ID	0.99
ntST 9	0.995	0.971	0.983	0.974	0.992	0.992	0.992	0.99	ID

The number, names, origin country, and accession no. of CMt strains included in each ntST are given in Fig. 1 footnote

**Fig. 2:**
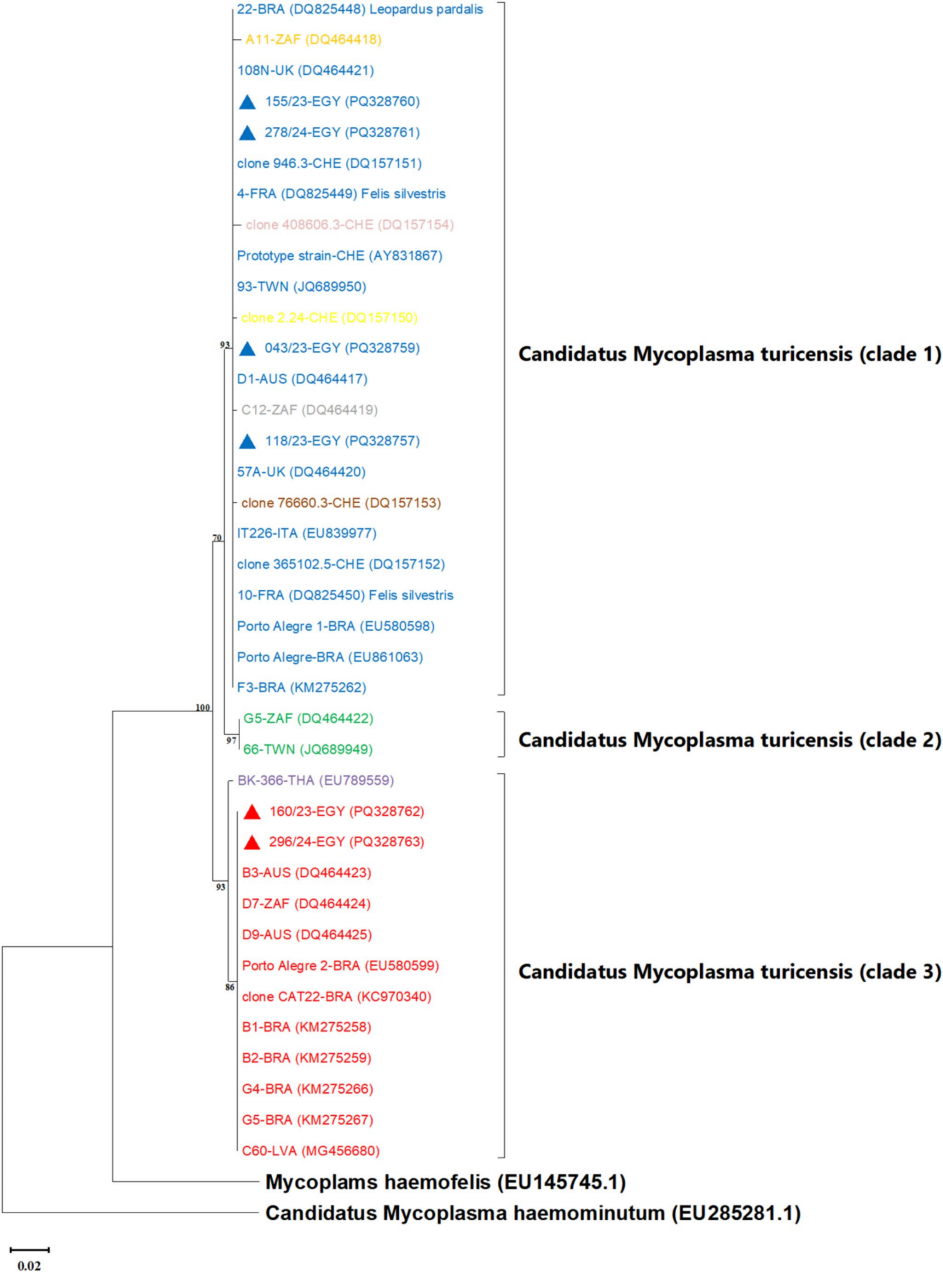
The maximum likelihood (ML) phylogenetic tree of 38 *Candidatus* Mycoplasma turicensis (CMt) partial *16s rRNA* gene sequences (425 nucleotides). The nucleotide substitution model used was the Kimura-2 parameters model. The ML tree was constructed by MEGA software version 11 and the robustness of the generated tree was inferred by bootstrap tests (1000 replicates); only bootstrap values > 70% were shown. The tree separated CMt strains into three distinct clades, and the Egyptian strains clustered within Clade 1 and Clade 3 as marked by blue and red triangles, respectively. Clade 1 contained six nucleotide sequence types (ntSTs) as follows: ntST 1 (blue font), ntST 5 (yellow font), ntST 6 (grey font), ntST 7 (brown font), ntST 8 (orange font), and ntST 9 (pink font). Clade 2 included one ntST (green font), and clade 3 involved two ntSTs, which are ntST 2 (red font) and ntST 3 (purple font). CMt Sequences included in this tree are identified by strain name followed by the country of origin (3-letter code) and accession number. Three strains were detected in wild felids (the host was indicated in these strains following the accession number) while other strains were detected in domestic cats. The 3-letter codes of different countries were as follows: AUS (Australia), BRA (Brazil), CHE (Switzerland), EGY (Egypt), FRA (France), ITA (Italy), LVA (Latvia), TWN (Taiwan), THA (Thailand), UK (United Kingdom), ZAF (South Africa). *Mycoplasma haemofelis* (EU145745) and *Candidatus* Mycoplasma haemominutum (EU285281) partial *16 S rRNA* gene sequences were added as outgroups. The scale bar represents the pairwise genetic distance

The CMt strains analyzed in this study formed three clades in the ML phylogenetic tree, supported by high bootstrap values; the Egyptian CMt strains were distributed across two of these three clades (Fig. 2). Furthermore, there was no geographic basis for this genetic separation.

## Discussion

This study sheds light on FHM epidemiology and CMt molecular characterization in cats in Egypt, a country with a scarcity of these data. The prevalence of FHM in Egyptian cats recorded in this study (16.2%) is lower than that reported in the previous study (20%), which examined a convenience sample of 100 client-owned cats in Cairo (Zarea et al. 2023). The larger sample size, wider study area, random sampling method, and inclusion of different cat types (client-owned and shelter-housed cats) in the current study might provide a more reliable prevalence estimation. Nonetheless, FHM detection rates in both studies fall within the well-known worldwide prevalence range (9–30%) (Aquino et al. 2014; Attipa et al. 2017; Barrs et al. 2010; Berzina et al. 2021; Ceylan et al. 2024a, b; Cetinkaya et al. 2016; Díaz-Regañón et al. 2018; Hoseinpoor et al. 2024; Jensen et al. 2001; Sarvani et al. 2018; Tanahara et al. 2010). This wide range may be attributed to differences in study areas, including climatic and vector burden variations, as well as in one or more components of the study design, such as sample size, sampling methods, diagnostic methods, and study population characteristics (e.g., health status, housing condition, and outdoor accessibility). These factors may positively or negatively affect the FHM detection rate within this range. In some studies, notable changes in these parameters resulted in prevalence rates highly divergent from this range. For example, a South African study focusing solely on anemic cats reported a high prevalence rate of 52% (Willi et al. 2006b), whereas a Canadian study limited to healthy, strictly indoor, client-owned cats found a markedly low prevalence of just 4% (Kamrani et al. 2008).

The FHM screening PCR used in this study cannot differentiate between Mhf and CMt due to their similar amplicon sizes. Although it can differentiate CMhm at 193 bp from Mhf/CMt at 170 bp, detecting whether CMhm + Mhf/CMt coinfections exist is difficult because of the very close amplicon sizes. Therefore, for proper typing of FHM and for detecting any form of coinfection, screening PCR-positive samples were subjected to a panel of three PCR methodologies, each specific for one FHM species. FHM typing by direct sequencing of the screening PCR-positive samples is not recommended, as this method amplifies preferentially bacteria with high loads in samples, reducing the chance of detecting coinfections (Aquino et al. 2014), particularly with CMt, which usually exhibits low-load bacteremia and predominantly infects cats already harboring other FHM species (Willi et al. 2006b).

The current study detected all FHM species and various coinfection forms, whereas the previous Egyptian study reported only single CMhm and Mhf infections, with neither CMt nor coinfections identified (Zarea et al. 2023). This discrepancy may be due to the use of direct sequencing for typing or the small sample size in the previous study. Consequently, the current research provides novel evidence for the presence of CMt and coinfections in cats in Egypt for the first time. The results of FHM typing in previous worldwide studies (*n* = 40) are summarized in Supplementary Table 1.

CMhm exhibited the highest prevalence among FHM species in this study similar to previous studies (Barker 2019; Ceylan et al. 2024a, b). The low virulence and efficient replication of CMhm in cats lead to a prolonged chronic carrier state (Tanahara et al. 2010). While this carrier state is described for all FHM species, it is most common with CMhm (Tasker 2010). These factors increase the odds of CMhm detection and explain its higher prevalence in this study and others (Supplementary Table 1). A notable exception was observed in one Canadian (Kamrani et al. 2008) and one Iranian study (Ghazisaeedi et al. 2014), where Mhf predominated over CMhm. In this study, CMhm was significantly associated with single infections rather than coinfections, unlike Mhf and CMt. This supports the ability of CMhm to establish infection independently, accounting for its higher prevalence rates. Similarly, in all previous studies listed in Supplementary Table 1, CMhm single infections predominated over coinfections, except in four studies conducted in Brazil (Aquino et al. 2014), Iran (Ghazisaeedi et al. 2014), Italy (Ravagnan et al. 2017), and South Africa (Willi et al. 2006b).

The low prevalence of Mhf and CMt in this study could be explained by the fluctuating nature and higher virulence of Mhf (Barker 2019), and the rapid elimination or tissue sequestration of CMt (Novacco et al. 2013; Tasker 2010). Both species were significantly associated with coinfections rather than single infections, suggesting their reliance on coinfection to establish themselves in cats, which may further explain their low prevalence. Mhf single infections were rarely observed in this study, consistent with findings from several other studies (Berzina et al. 2021; Cetinkaya et al. 2016; Gentilini et al. 2009; Jenkins et al. 2013; Martínez-Díaz et al. 2013; Sarvani et al. 2018; Tanahara et al. 2010). CMt single infections were not identified in this study population, aligning with findings from many studies that detected CMt exclusively in coinfection form (Aquino et al. 2014; Barrs et al. 2010; Ceylan et al. 2024a, b; Díaz-Regañón et al. 2018; Fujihara et al. 2007; Just and Pfister 2007; Roura et al. 2010).

This study identified some coinfection forms of FHM, suggesting the absence of cross-protection between different FHM species (Tasker et al. 2018). Such coinfections may enhance the infectivity and pathogenicity of FHM species; indeed, several studies have reported more severe disease in coinfected cats than in singly infected ones (Baumann et al. 2015; Willi et al. 2006a).

Anemia was significantly associated with higher overall FHM-PCR positivity (OR = 3.5), consistent with other studies (Jensen et al. 2001; Sarvani et al. 2018). FHM induces anemia primarily through extravascular hemolysis (Barker 2019). In particular, Mhf is recognized as a major infectious cause of feline anemia (Tasker et al. 2018). Anemia was not identified as a predictor of single CMhm infection in this study, reflecting the lower pathogenicity of this species, which further accounts for its higher prevalence. CMhm-associated disease often occurs in immunosuppressed cats or those coinfected with other FHM species (Barker 2019). Supporting this, HCT, RBC, and Hb levels in single CMhm-infected cats were not significantly different from those in the control group. In contrast, these hemogram parameters were significantly lower in overall FHM-infected cats compared to non-infected ones, although they remained within the lower reference ranges, possibly due to the inclusion of many chronically infected cats.

In this study, sex was significantly associated with higher overall FHM infection (OR = 10) and single CMhm infection (OR = 8.2) rates, similar to previous studies (Aquino et al. 2014; Berzina et al. 2021; Ceylan et al. 2024a, b; Sarvani et al. 2018; Tanahara et al. 2010). The sexual behavior of male cats drives them to roam outside and engage in fights, which is identified as a possible FHM transmission route (Museux et al. 2009) and likely a major route, more significant than flea transmission (Moore et al. 2024).

The high nucleotide identity percentages of CMt sequences, including those obtained in this study (Table 5), indicate low *16 S rRNA* gene diversity among different CMt strains. On phylogenetic analysis, most Egyptian and reference strains, including the prototype strain, clustered into the first clade, suggesting these strains may have advantages in replication efficiency, persistence, or survival compared to strains of other clades. A striking finding is that the partial *16 S rRNA* gene-based tree topology obtained here was similar to the nearly full gene-based one reported previously (Aquino et al. 2014), indicating that this gene region is phylogenetically informative and suitable for conducting phylogenetic analyses. Interestingly, this phylogenetic separation did not follow any geographic pattern (Aquino et al. 2014; Willi et al. 2006b), precluding the use of this gene-based phylogeny to predict potential introduction sites during epidemiological investigations.

A significant limitation of this study was the absence or low number of cats with various FHM infection types (CMt and Mhf single infections, as well as CMhm + CMt and Mhf + CMt coinfections), precluding the ability to investigate risk factors and hematological abnormalities associated with these infection types. A second limitation was that most samples were taken during the winter and spring seasons of the study population; therefore, we observed only a few cats infested with fleas, prohibiting the ability to study the role of fleas in disease transmission. A further limitation was the low number of client-owned cats included in this study compared to shelter-housed ones, which might lead to a potential distortion of the statistical analysis.

## Conclusions

The current research updates the limited data regarding the epidemiology and molecular characterization of FHM in Egypt, enhancing our ability to implement more efficient control strategies. The three major FHM species were identified in the study population with an overall prevalence of 16.2%, aligning with the worldwide prevalence range. CMhm had the highest prevalence and was significantly associated with single infections, supporting the conclusion that CMhm can establish infections independently. CMt and several forms of FHM coinfections were detected for the first time in Egypt, reflecting the value of using an FHM species-specific PCR panel in epidemiological studies. Male cats had a significantly higher FHM prevalence rate, supporting the role of aggressive interaction in disease transmission. Partial sequencing was successful for all CMt strains detected in this study, marking the first appearance of Egyptian CMt sequences in GenBank.

## Electronic supplementary material

Below is the link to the electronic supplementary material.

Supplementary Material 1

Supplementary Material 2

## Data Availability

No datasets were generated or analysed during the current study.
