# Supplementary material for: Typing of hemotropic Mycoplasma in Egyptian cats: first detection and phylogenetic analysis of Candidatus Mycoplasma turicensis
Source: Vet Res Commun. 2025 Mar 11;49(3):136. doi: 10.1007/s11259-025-10693-0 (PMC11897094; doi:10.1007/s11259-025-10693-0)
Supplement: Supplementary file 2 — Supplementary Material 2 [file 11259_2025_10693_MOESM2_ESM.docx]

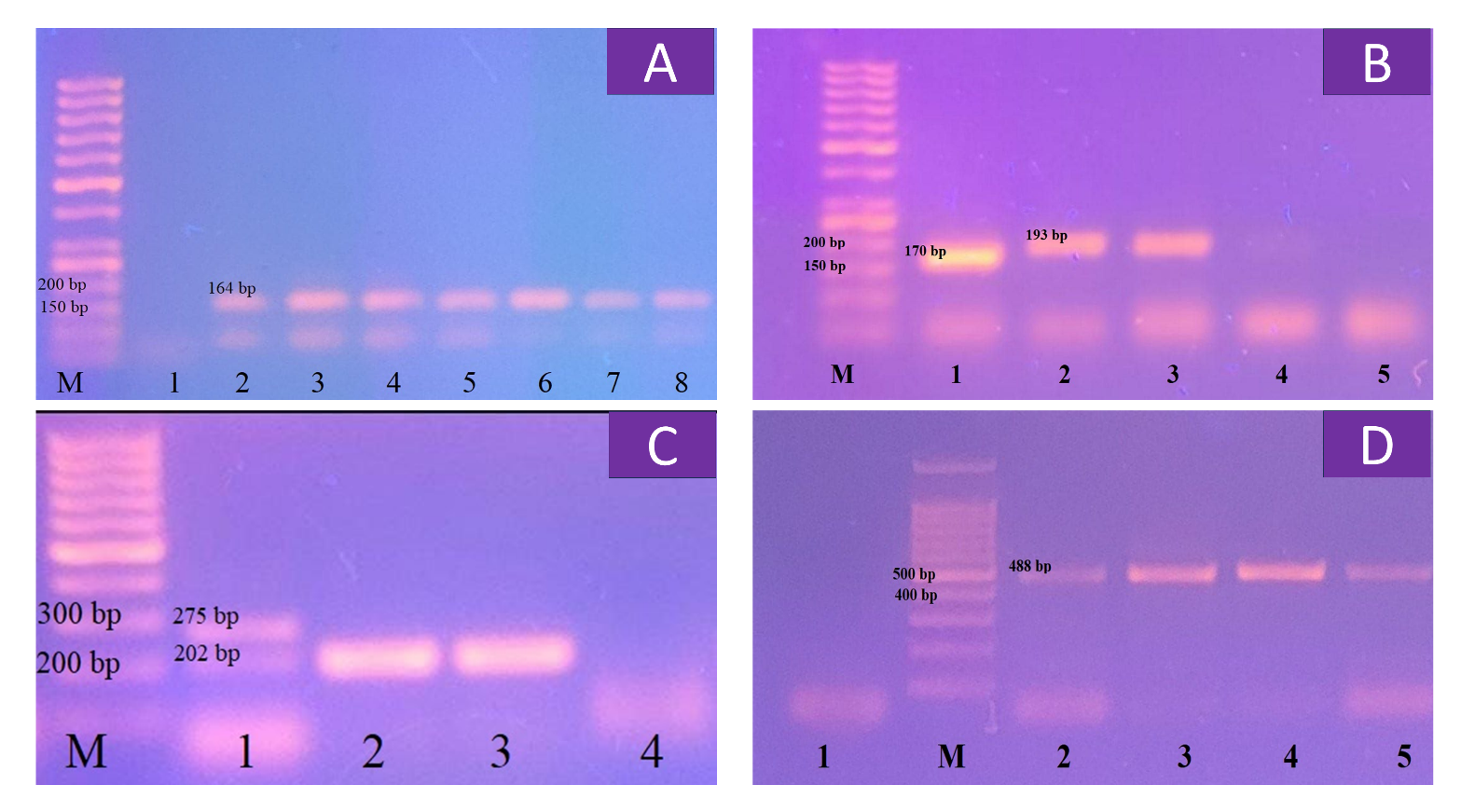
**Supplementary Figure 1.** Ethidium bromide-stained agarose gel pictures of all conventional PCR methodologies used in this study. (A) Internal control (GAPDH) PCR; samples showing 164 bp amplicons were considered positive (M: 50 bp-ladder; 1: negative control; 2-8: positive samples). (B) FHM screening PCR; samples showing 170 bp were considered positive for Mhf or CMt while samples showing 193 bp amplicons were considered positive for CMhm (M: 50 bp-ladder; 1: sample positive for Mhf/CMt; 2-4: samples positive for CMhm; 5: negative control). (C) Multiplex PCR specific for either Mhf or CMhm; samples showing 202 bp were considered positive for CMhm while samples showing 275 bp amplicons were considered positive for Mhf (M: 100 bp-ladder; 1: sample positive for both Mhf and CMhm; 2-3: samples positive for CMhm; 4: negative control). (D) PCR specific for CMt; samples showing 488 bp amplicons were considered positive (M: 100 bp-ladder; 1: negative control; 2-5 positive samples). All screening and species-specific FHM PCR methodologies partially amplify the *16S rRNA gene*.

Abbreviations (**CMhm**: *Candidatus* Mycoplasma haemominutum; **CMt**: *Candidatus* Mycoplasma turicensis; **FHM**: feline hemotropic Mycoplasma; **Mhf**: *Mycoplasma haemofelis*).
